# Acoustic radiation force impulse (push pulse)-induced lung hemorrhage: investigating the effect of ultrasound contrast agent in rabbits

**DOI:** 10.1007/s10396-024-01510-3

**Published:** 2024-11-16

**Authors:** Noriya Takayama, Hideki Sasanuma, Kazuma Rifu, Naotaka Nitta, Iwaki Akiyama, Nobuyuki Taniguchi

**Affiliations:** 1 Department of Clinical Laboratory Medicine, Jichi Medical University, 3311-1 Yakushiji, Shimotsuke, Tochigi 329-0498 Japan; 2 Division of Gastroenterological, General and Transplant Surgery, Department of Surgery, Jichi Medical University, Shimotsuke, Tochigi Japan; 3 Health and Medical Research Institute, National Institute of Advanced Industrial Science and Technology (AIST), Tsukuba, Ibaraki Japan; 4 Medical Ultrasound Research Center, Doshisha University, Kyotanabe, Kyoto Japan

**Keywords:** Acoustic radiation force impulse, Ultrasound contrast agent, Lung, Hemorrhage, Mechanical index

## Abstract

**Purpose:**

Acoustic radiation force impulse (ARFI) elastography and contrast-enhanced ultrasonography (CEUS) are emerging techniques that are becoming common in ultrasound examinations. We previously reported that ARFI (push pulse) induced lung hemorrhage in rabbits, indicating that greater risks are associated with ARFI than with conventional ultrasound. In this study, we assessed the risk of lung hemorrhage under a combination of ARFI elastography and CEUS, considering potential exacerbation of ARFI-induced lung hemorrhage as a result of the ultrasound contrast agent (UCA) used in CEUS.

**Methods:**

Twenty-three rabbits were divided into non-UCA and UCA groups. ARFI exposure parameters were set at six mechanical index (MI) levels (0.29, 0.45, 0.60, 0.88, 1.0, 1.39) in non-UCA groups and five MI levels (0.29, 0.66, 0.88, 0.97, 1.25) in UCA groups. Lung exposure was performed bilaterally through the intercostal space in each rabbit. Lung damage was assessed through macroscopic and microscopic observation post euthanasia.

**Results:**

Lung hemorrhage was detected at MI_0.3_ levels of 0.88 or higher. Logistic regression analyses showed that MI_0.3_ was a statistically significant factor for occurrence of lung hemorrhage in both non-UCA and UCA groups, and the MI_0.3_ threshold (ED_05_) for inducing lung hemorrhage was 0.68 and 0.71, respectively. However, multivariate logistic regression and linear regression analyses across all samples indicated that UCA did not significantly affect the occurrence or area of lung hemorrhage.

**Conclusion:**

This study demonstrates that UCA does not significantly worsen ARFI-induced lung hemorrhage in terms of occurrence or severity. However, risks and benefits of ARFI elastography on the lung should be considered, irrespective of UCA administration.

## Introduction

There have been no reports of harmful effects induced by diagnostic ultrasound in humans. However, lung tissue is known to be fragile under ultrasound exposure. There have been many reports of ultrasound-induced lung hemorrhage at the diagnostic level in animal experiments [1]. Furthermore, ultrasound-induced lung hemorrhage has been demonstrated using actual clinically applied imaging modalities in various modes, such as B mode or Doppler modes in rats [2]. Recently, new ultrasound modalities such as acoustic radiation force impulse (ARFI) elastography and contrast-enhanced ultrasonography (CEUS) have been introduced into clinical settings and have become common. It is important to assess the safety of these new techniques as the conditions of ultrasound exposure differ from those used in conventional modalities. Several studies have evaluated the bioeffects of these new ultrasound modalities.

ARFI elastography utilizes focused, high-intensity ultrasound with a longer pulse duration (PD) compared to conventional ultrasound modalities. The term “ARFI” can be used to denote ultrasound with acoustic radiation force utilized for the purpose of ARFI elastography, which is also referred to as “push pulse.” Because the safety of ARFI elastography is not well known, we conducted an animal study using rabbits and were the first to report that ARFI (PD, 10 ms) induces lung hemorrhage [3]. Subsequently, we investigated the effect of the peak rarefactional pressure amplitude (PRPA) or mechanical index (MI) of the push pulse (PD, 0.3 ms) on lung hemorrhage. In our study, the threshold to induce lung hemorrhage in rabbits was estimated as an MI_0.3_ (attenuation factor: 0.3 dB/cm/MHz) of 0.5. [4]. There have been no reports of lung damage caused by ultrasound in humans, and larger animals tend to have a higher threshold for lung hemorrhage [5]. However, lung hemorrhage has been reported in rats using clinically applied ARFI elastography [6, 7]. These results suggest that ARFI elastography could be associated with a greater risk of lung hemorrhage than conventional diagnostic ultrasound modalities.

In 2004, the European Federation of Societies for Ultrasound in Medicine and Biology (EFSUMB) initiated the use of CEUS administered with an ultrasound contrast agent (UCA) consisting of microbubbles for creating contrast imaging [8]. Perfluorobutane, a commercially available second-generation UCA, is commonly used to evaluate liver and breast tumors in Japan. Although CEUS is considered safe because MI is generally regulated below 0.3, cavitation, one of the main mechanical effects of ultrasound, is assumed to occur in the presence of microbubbles, potentially leading to microvascular disruption in the tissue as a bioeffect [9].

Therefore, it is possible that the bioeffect of ARFI elastography is exacerbated by the use of UCA, although it is uncommon for ARFI elastography to be performed shortly after CEUS or simultaneously. We have also reported that the presence of UCA facilitates ARFI by inducing arrhythmias in rabbits [10]. Therefore, we hypothesized that ARFI under UCA administration could exacerbate lung hemorrhage or lower the threshold, leading to the possibility of lung hemorrhage even in humans in clinical settings. However, no studies have investigated the effects of ARFI in combination with UCA administration in the lungs. Therefore, we investigated whether UCA could exacerbate ARFI-induced lung hemorrhage. This study aimed to assess the risk of lung injury associated with ARFI elastography after UCA administration. We conducted an animal experiment using an ARFI exposure system capable of emitting the push pulse equivalent to ARFI elastography and perfluorobutane.

## Materials and methods

### Animal model and ethical approval

In total, 23 male Japanese white rabbits (16 to 18 weeks old) with an average weight of 3.0 ± 0.2 kg were utilized for this study. The experiment was conducted as part of joint research on the heart and lungs [4, 10] with respect to the assessment of the safety of ARFI elastography. The local Institutional Care and Animal Use Committee approved the study protocol (approval number: 17233, 20,162).

### Acoustic radiation force impulse (ARFI) exposure system

We used an ARFI exposure system (Front-End Technology, Nagano, Japan) equipped with a linear probe. The system was connected to a personal computer for operation and a B-mode imaging monitor.

**Fig. 1:**
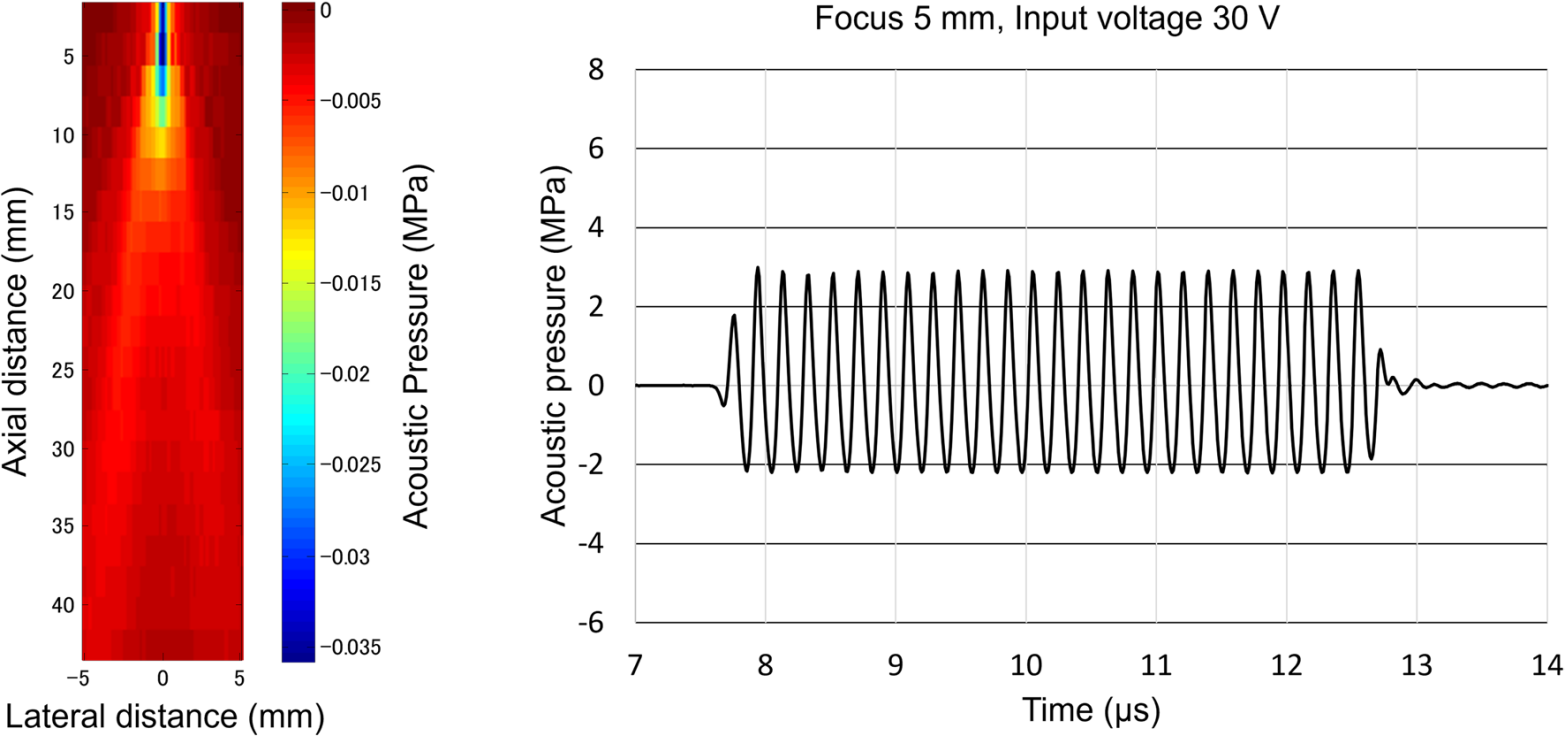
Acoustic profile of the push pulse utilized in this experiment, measured in degassed water using a needle hydrophone. Left: Negative sound pressure distribution of the push pulse (axial beam width, axial distance from 2.5 to 8.5 mm*; lateral beam width, 0.7 mm; full width at half maximum, respectively). *Although the axial beam width was approximately 8 mm, assuming twice the one-sided range of the full width at half maximum, no measurement was performed at an axial distance of < 2.5 mm to prevent the hydrophone from being damaged. Right picture: Representative acoustic pressure waveform at an input voltage of 30 V (MI_0.3_ = 0.88). Note that the hydrophone was placed at the peak point of the sound pressure distribution

A controller (RSYS-0004; Microsonic, Tokyo, Japan) and power amplifier (Array Transmitter SYS-0013; Microsonic) were used in this system. Push pulses were emitted with a driving signal frequency of 5.2 MHz from the focused linear probe (pitch, 0.2 mm; 128 elements with no apodization; linear array; bandwidth, 4–15 MHz). The acoustic profile of the push pulse was measured in degassed water using a needle-type hydrophone (HNR-0500; Onda Corporation, Sunnyvale, CA, USA). The negative acoustic pressure distribution (axial beam width, axial distance from 2.5 to 8.5 mm; lateral beam width, 0.7 mm; full width at half maximum) and a representative acoustic pressure waveform at the focal point of 5 mm (16 of 128 elements were used), where an input voltage of 30 V was applied, are shown in Fig. 1.

The exposure settings were equivalent to those of ARFI elastography in clinical use. PD was set to 0.3 ms according to typical modalities with a push pulse of 0.05–1 ms [11]. The number of push pulse emissions was set to 30 because > 10 measurements are recommended in clinical practice; the time interval was set to 3 s because common modalities have freeze durations for a few seconds between each measurement [12].

The MI was determined to be proportional to the value of the derated PRPA, as per the Basic Concepts of Safety of Diagnostic Ultrasonic Equipment [13, 14], using the following equation:

$${\mathrm{MI}}_{{0.{3}}} = p_{r.3} /\sqrt{f_{c}}$$

Here, *p*_*r.3*_ (in MPa) represents the derated PRPA, which is the water-based peak negative acoustic pressure derated by 0.3 dB/cm-MHz at the location where the derated pulse intensity integral is considered the maximum and *f*_*c*_ is the center frequency (in MHz).

### UCA

**Fig. 2:**
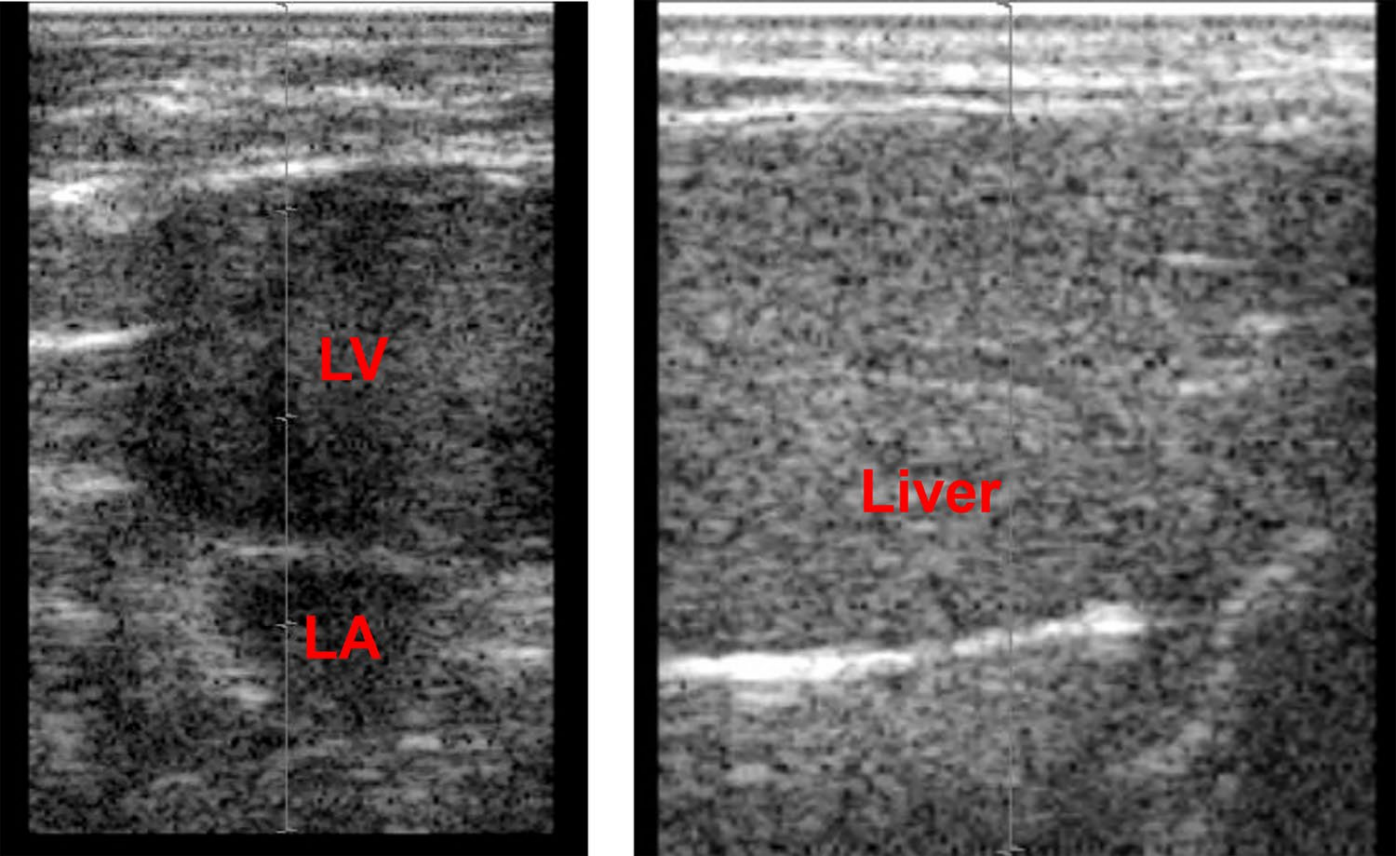
The presence of UCA in the tissue was confirmed under B-mode observation of the heart and the liver. *UCA* ultrasound contrast agent. Left: Sonazoid was detected as high-echoic blood flow in the cardiac chambers. *LV* left ventricle, *LA* left atrium. Right: Sonazoid was detected as high-echoic liver parenchyma where Sonazoid was taken up by Kupffer cells

UCA is supposed to be administered intravenously during CEUS. UCA contains microbubbles that oscillate when exposed to ultrasound, enhancing the vascular contrast of tissue. A second-generation UCA, Sonazoid (perfluorobutane; Daiichi-Sankyo / GE Healthcare), was used in this experiment. A bolus injection of 0.8 µl Sonazoid, which is approximately double the clinical dose, was administered 2 min before ARFI exposure because the time to maximum concentration of Sonazoid is approximately 2 min. After the administration of Sonazoid, its presence in the tissue was confirmed under B-mode observation of the heart and the liver. Sonazoid was detected as high echoic blood flow in the cardiac chamber and as high echoic liver parenchyma where Sonazoid was taken up by Kupffer cells (Fig. 2).

### Animal experiment procedure

**Fig. 3:**
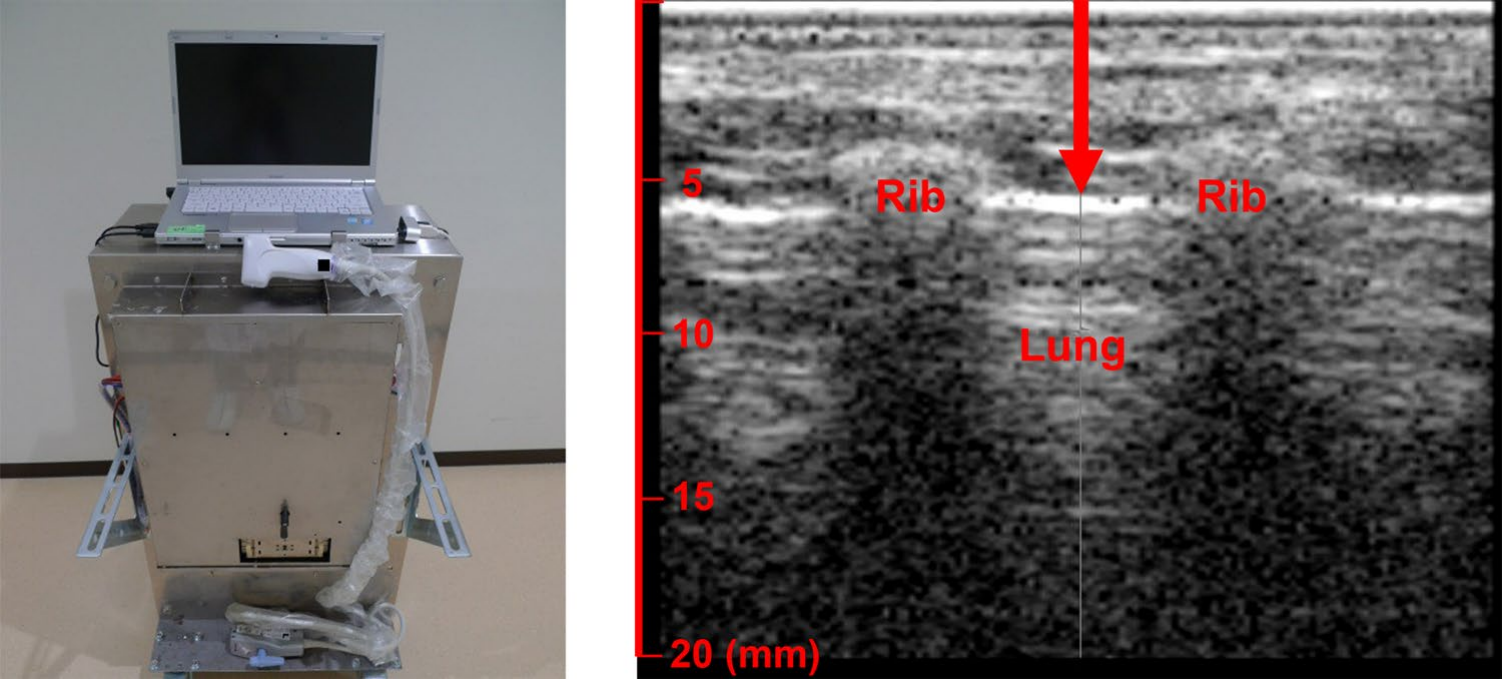
ARFI exposure in this experiment. Left: ARFI exposure system equipped with a linear probe and connected to a personal computer to display B-mode imaging. Right: The red arrow shows the push pulse pathway between ribs from the skin to the lung surface. The arrowhead points to the pleural line (high-echoic line) at a focal point of 5 mm. *ARFI* acoustic radiation force impulse

Premedication with anesthetic was initiated with an intramuscular injection comprising 200 µg/kg medetomidine, 0.15 mg/kg butorphanol, and 0.15 mg/kg midazolam. Each rabbit was placed in the supine position. General anesthesia was induced by sevoflurane inhalation. An electrocardiograph and pulse oximeter were attached to the rabbit to monitor heart rate, respiratory rate, electrocardiogram, and oxygen saturation during the procedure. A tracheotomy was performed to insert a tracheal tube, and ventilation was introduced to control breathing during the experiment. Both the right and left lateral thoracic regions were shaved and depilated, and a linear probe was positioned to identify the appropriate location in the intercostal space for ARFI exposure under B-mode imaging. After the targeted lung location was defined, an intravenous injection of 10 mg/kg propofol was administered to stop respiration and fluctuations in the area. B-mode imaging was turned on solely to locate an appropriate target area and confirm the focal point on the lung surface. Slight adjustment for the positioning of the probe using ultrasound gel or manual compression was performed to set the focus on the lung surface, although the distance from the skin surface to the lung surface through the intercostal space was approximately 5 mm in the rabbits in this experiment (Fig. 3). Subsequently, B-mode imaging was turned off to reduce its effect on the lungs, and push pulses were emitted under the following conditions:

Twelve rabbits were exposed to ARFI without UCA: six groups of two rabbits each, and a sham group of one rabbit (non-UCA group). Six input-voltage steps were applied to the six groups. PRPA_0.3_ and MI_0.3_ of each group varied as follows: (1) 0.66 MPa (MI, 0.29), (2) 1.03 MPa (MI, 0.45), (3) 1.37 MPa (MI, 0.60), (4) 2.01 MPa (MI, 0.88), (5) 2.32 MPa (MI, 1.0), and (6) 3.17 MPa (MI, 1.39). Nine rabbits were exposed to ARFI with UCA: five groups of two rabbits each, and a sham group of one rabbit (UCA group). Five input-voltage steps were applied to five groups. PRPA_0.3_ and MI_0.3_ of each group varied as follows: (7) 0.66 MPa (MI, 0.29), (8) 1.51 MPa (MI, 0.66), (9) 2.01 MPa (MI, 0.88), (10) 2.21 MPa (MI, 0.97), and (11) 2.85 MPa (MI, 1.25). The range of MI was set at the input-voltage steps from 10 to 50 V. The minimum setting of MI 0.29 (10 V) was selected, assuming it to be approximately half of the threshold to induce lung hemorrhage, which was MI 0.5 in our previous experiment. Moreover, the maximum setting of MI 1.39 (50 V) was chosen on the basis of the risk of damage to the probe due to higher input voltage. The reason for the different MI between non-UCA and UCA groups was that there were slight changes between the initial measurement results of sound pressure and subsequent results owing to aging of the ARFI exposure system. (Note that this experiment was interrupted at the end of 2019 owing to the COVID-19 pandemic and was restarted in May 2022. Therefore, there was a hiatus of 2 years and 5 months during the experiment, necessitating the second set of measurements to ascertain aging changes on the resumption of the experiment. Experiments for groups (1)–(7) and (9) had been conducted before the hiatus, while those for groups (8), (10), and (11) were performed after the hiatus; thus, results of the MI measurement resulted in differences.) Push pulse emission was initially performed in one area of the right lung followed by the left lung. Two samples were obtained from each rabbit, as one point of the right and left lungs was exposed for each rabbit.

After ARFI exposure, the rabbits were euthanized under anesthesia, and a thoracotomy was performed to verify the occurrence of gross damage on the bilateral lung surface corresponding to the area of ARFI exposure. Both lungs with the heart attached to the trachea were removed. The lungs were inflated using a bag valve mask through the tracheal tube inserted into the trachea to ensure clear visualization of the lesion and to measure the long and short diameters of the lesion in the inflated lung. Subsequently, digital photographs of the inflated lungs were obtained, and image analysis software (Photoshop 2022; Adobe Inc., San Jose, CA, USA) was used to calculate the lesion area; this was performed by tracing the border of the lesion and counting the number of pixels inside the demarcated region, which was converted into the lesion area based on the measured long diameter of the lesion. Each lesion area was cut into cross-sections and prepared for pathological studies after fixation with formalin instillation. Histological slides were microscopically analyzed under hematoxylin and eosin staining. The area, including the pleura and surrounding lung tissue, was examined to confirm the pathological findings of the lesion in the lung.

### Statistical analyses

All statistical analyses were performed using the R software (R Foundation for Statistical Computing, Vienna, Austria; https://www.R-project.org/). A logistic regression model was applied to the samples in the non-UCA and UCA groups to determine the dependence of lung hemorrhage occurrence on MI_0.3_ in each group. The explanatory variable was the value of MI_0.3_, and the objective variable was the occurrence of lung hemorrhage. These estimates were transformed to yield a threshold of MI_0.3_ associated with a 5% probability of lung hemorrhage occurrence (ED_05_: effective dose of an intervention that produces a specific effect in 5% of animals administered the dose) in each group. Furthermore, a multivariate logistic regression model was applied to all samples to identify the effect of UCA on the incidence of lung hemorrhage. The explanatory variables were the value of MI_0.3_ and the use of UCA, and the objective variable was the occurrence of lung hemorrhage. Additionally, a linear regression model was applied to samples in the non-UCA and UCA groups to ascertain the dependence of the lung hemorrhage area on MI_0.3_ in each group. The explanatory variable was the value of MI_0.3_, and the objective variable was the size of the lung hemorrhage area (mm^2^). Similarly, multivariate linear regression was conducted for all samples to investigate the effect of UCA on the size of the lung hemorrhage area. The explanatory variables comprised the value of MI_0.3_ and the use of UCA, while the objective variable was the size of the lung hemorrhage area. The size of the lung hemorrhage area was defined as zero if no lesions were observed. By including the different MI values between the non-UCA and UCA groups as continuous variables, these statistical analyses could detect statistical differences in objective variables between the two groups.

## Results

Throughout ARFI exposure, no significant changes were noted in the electrocardiogram and oxygen saturation monitor, and no abnormal clinical findings were observed in the rabbits. Neither hemothorax nor pneumothorax was observed in the thoracotomy findings. A red spot corresponding to the area of ARFI exposure was observed on the surface of the lungs. Microscopic examination revealed extravascular red blood cells and confirmed alveolar hemorrhage at each red spot. No pathological differences were observed between the non-UCA and UCA groups.

**Fig. 4:**
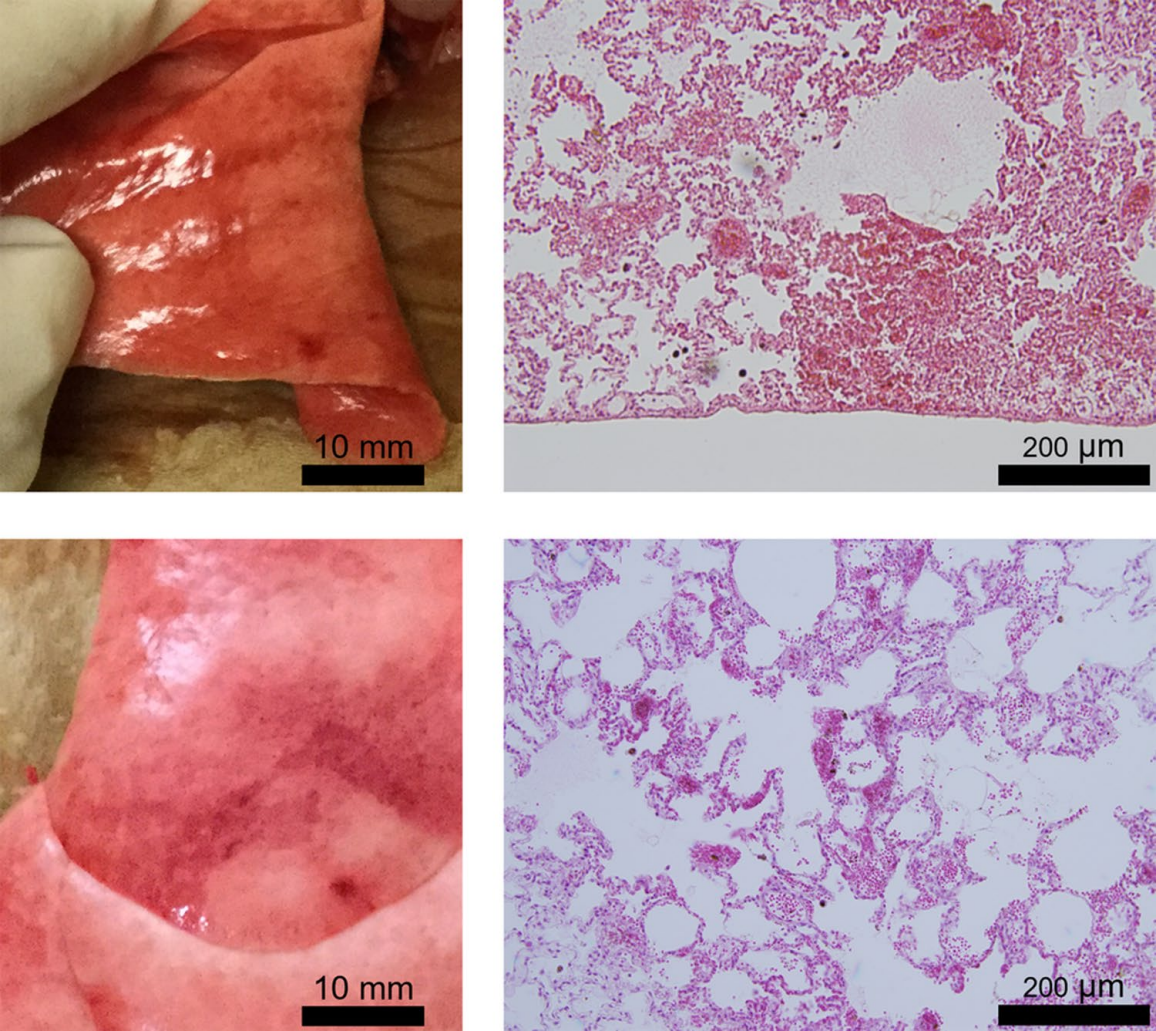
Typical lung hemorrhage induced by push pulse in the non-UCA group and UCA group at an MI_0.3_ of 0.88 in this experiment. *UCA* ultrasound contrast agent, *MI* mechanical index. Left: Macroscopic images showing a red spot in the non-UCA group (upper picture) and UCA group (lower picture). Scale bar: 10 mm. Right: Microscopic images of each red spot in the non-UCA group (upper picture) and UCA group (lower picture) reveal extravascular erythrocytes in the alveolar tissue. These histological findings were consistent with pulmonary alveolar hemorrhage but did not indicate a pathological difference between the non-UCA and the UCA groups. Scale bar: 200 µm

The results of lung hemorrhage occurrence and mean area at each MI_0.3_ in the non-UCA and UCA groups are shown in Table 1(a) and (b), respectively. Lung hemorrhage was detected in groups (4)–(7) in the non-UCA groups and (9)–(11) in the UCA groups, where the value of MI_0.3_ was 0.88 or higher. The occurrence of lung hemorrhage in all samples was as follows: (1) 0/4, (2) 0/4, (3) 0/4, (4) 1/4, (5) 1/4, and (6) 3/4 in the non-UCA groups, and (7) 0/2, (8) 0/4, (9) 1/4, (10) 1/4, and (11) 3/4 in the UCA groups (Table 1 and Fig. 4). Logistic regression analyses showed that MI_0.3_ was a statistically significant factor for the occurrence of lung hemorrhage in both the non-UCA (*p* < 0.05) and UCA groups (*p* < 0.05). The coefficients derived from these analyses provided estimates for MI_0.3_ associated with a 5% probability of lung hemorrhage occurrence, considering that the threshold was calculated as an MI_0.3_ of 0.68 in the non-UCA group and 0.71 in the UCA group. However, the multivariate logistic regression analysis of all samples showed that the use of UCA was not a significant factor in the occurrence of lung hemorrhage (*p* $=$ 0.71).

**Table 1:** Summary of lung hemorrhage occurrence and mean area in each non-UCA group (a) and UCA group (b)

(a) Non-UCA groups					
Group	Number of samples*	PRPA_0.3_** (MPa)	MI_0.3_**	Occurrence	Mean area*** (mm^2^)
(1)	4	0.66	0.29	0 / 4	0
(2)	4	1.03	0.45	0 / 4	0
(3)	4	1.37	0.60	0 / 4	0
(4)	4	2.01	0.88	1 / 4	3.0 ± 3.0
(5)	4	2.32	1.00	1 / 4	4.5 ± 4.5
(6)	4	3.17	1.39	3 / 4	20.5 ± 8.7
Sham	2	0	0	0 / 2	0

(b)UCA groups					
Group	Number of samples*	PRPA_0.3_** (MPa)	MI_0.3_**	Occurrence	Mean area*** (mm^2^)
(7)	2	0.66	0.29	0 / 4	0
(8)	4	1.51	0.66	0 / 4	0
(9)	4	2.01	0.88	1 / 4	2.0 ± 2.0
(10)	4	2.21	0.97	1 / 4	4.0 ± 4.0
(11)	4	2.85	1.21	3 / 4	11.3 ± 4.6
Sham	2	0	0	0 / 2	0

A logistic regression model showed that MI_0.3_ was a statistically significant factor for the occurrence of lung hemorrhage in both the non-UCA (*p* < 0.05) and UCA groups (*p* < 0.05). However, multivariate logistic regression analysis of all samples showed that the use of UCA was not a significant factor in the occurrence of lung hemorrhage (*p* = 0.71)

*UCA* ultrasound contrast agent, *PRPA* peak rarefactional pressure amplitude, *MI* mechanical index

^*^Two samples were obtained from each rabbit exposed to a push pulse in the right and left lungs

^**^PRPA_0.3_ assumed a 0.3 dB/cm-MHz deration scheme, from which MI_0.3_ is calculated

^***^Mean area of the lesion ± standard error

**Fig. 5:**
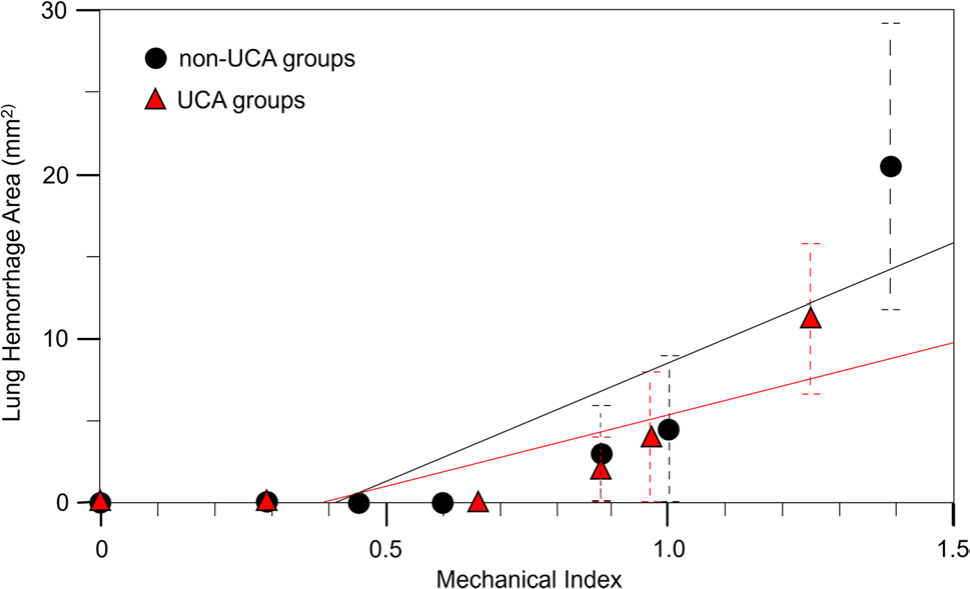
Plots of lung hemorrhage mean area with standard error bars in all groups. Linear regression lines are shown for non-UCA groups and UCA groups, respectively. *UCA* ultrasound contrast agent. Linear regression models showed a positive correlation between MI_0.3_ and the lung hemorrhage area in the non-UCA (*p* < 0.05) and the UCA (*p* < 0.05) groups. However, multivariate linear regression analysis of all samples showed that UCA was not a significant risk factor for the lung hemorrhage area (*p* = 0.43). *MI* mechanical index

The mean lesion areas in each group were as follows: (4) 3.0 mm^2^, (5) 4.5 mm^2^, and (6) 20.5 mm^2^ in the non-UCA groups, and (9) 2.0 mm^2^, (10) 4.0 mm^2^, and (11) 11.3 mm^2^ in UCA group (Table 1 and Fig. 5). Linear regression analyses showed that MI_0.3_ was a statistically significant factor for the size of the lung hemorrhage area in both the non-UCA (*p* < 0.05) and UCA groups (*p* < 0.05). However, similar to lung hemorrhage occurrence, multivariate linear regression analysis showed that UCA was not a significant factor for lung hemorrhage area (*p* $=$ 0.43).

## Discussion

In this study, we conducted an animal experiment aimed at assessing the risk of tissue damage resulting from ultrasound exposure on the assumption that ARFI elastography could be performed after CEUS, because this combination could result in unexpected damage to the human body.

This is the first study to evaluate the effect of UCA on ARFI-induced lung hemorrhage. We have already demonstrated that lung hemorrhage can be induced by ARFI at a lower threshold than conventional ultrasound [4]. Therefore, there could be a greater risk of lung hemorrhage if ARFI-induced lung hemorrhage is exacerbated by UCA administration. However, the results of this study demonstrated that UCA was not a significant factor affecting ARFI-induced lung damage, whereas PRPA was a strong factor affecting aggravated lung hemorrhage.

The mechanism underlying ultrasound-induced lung hemorrhage has not yet been elucidated. We assumed that the mechanical effect, rather than the heat effect, is primarily responsible, given that the push pulse used in this study only raises temperatures by a few degrees Celsius, as demonstrated in our previous study [15]. Cavitation is believed to play a major role in this mechanical effect; under the safety regulations governing the clinical use of diagnostic ultrasound, this phenomenon generally occurs only in the presence of microbubbles. The UCA contains microbubbles, which amplify the mechanical effect. Our findings that UCA has no additional effect on lung damage could indicate that the anatomical structure of the lung itself contributes more significantly to ultrasound-induced damage than the presence of microbubbles. Lung tissue consists of many sets of gas-containing alveoli with a vast number of minute air spaces. This characteristic tissue structure is thought to play a more significant role in causing damage than the presence of microbubbles in the tissue.

The US Food and Drug Administration guidance sets an MI limit of 1.9 for diagnostic ultrasound modalities, and this same regulation applies to ARFI elastography, which emits push pulses different from conventional ultrasound. However, the safety of ARFI elastography in terms of MI has not been sufficiently assessed. In a previous study, we reported that push pulses induced lung hemorrhage in rabbits at the clinical level and that the extent of damage depended on the MI value [4]. CEUS is a recent technique that has been used to enhance the contrast of blood flow in tissue by intravenously injecting UCA. Microbubbles present in UCAs are known to exacerbate the bioeffects on the heart [10]. The Japan Society of Ultrasonics in Medicine announced that ARFI and CEUS should not be performed simultaneously. Therefore, we hypothesized that ARFI-induced lung hemorrhage could be exacerbated by UCA administration. The result of our experiment showed that the estimated threshold (ED_05_) of MI_0.3_ to induce lung hemorrhage was 0.68 in the non-UCA group and 0.71 in the UCA group. Although this study did not demonstrate the effect of UCA on ARFI-induced lung hemorrhage, lung tissue damage could occur within the regulation value of MI, regardless of UCA administration. Therefore, we suggest that all examiners should be aware of the risk of lung hemorrhage induced by ARFI elastography. When applying ARFI elastography, especially to lung tissue, the risks and benefits should be carefully considered. Additionally, caution should be exercised when applying ARFI elastography to organs that are adjacent to the lungs and are the main target of the examination, such as the liver or breast, because incidental exposure to ARFI is possible.

### Limitations

This study was performed to assess the clinical risk of lung hemorrhage due to ARFI elastography under UCA administration. However, this study was subject to the following limitations:

First, ARFI elastography, which is currently used in clinical settings, typically contains baseline B-mode pulses and shear-wave imaging pulses in addition to several push pulses in a single measurement, whereas the ARFI exposure system in this experiment emitted only pure push pulses. Two reports evaluated the risk of lung hemorrhage in rats using clinically applied ARFI elastography, which includes these three types of pulses [6, 7]. It is unknown whether the effect of these ultrasound pulses other than the push pulse under UCA administration plays an unexpected role in exacerbating lung damage. Future studies should investigate the risk of ARFI-induced lung hemorrhage under UCA using clinically applied modalities.

Second, the presence of UCA in the lung tissue, which was the focus of the push pulse in this experiment, was not directly confirmed. The assumption that UCA was present in the lung tissue was based on its detection in the heart and liver under B-mode imaging. However, direct detection of the UCA at the lung surface under B-mode imaging was difficult due to the interference caused by alveolar gas reflex. Real-time observation of the contrast would have provided the most reliable evidence for the presence of UCA during the experiment. Future studies should assess methods to confirm the presence of UCA in the lung tissue as proving the presence of UCA at the lung surface was a primary concern in this study.

Third, the results of this study were obtained from animal experiments using rabbits. It is difficult to extrapolate these results to humans in clinical settings because larger animals tend to have a higher threshold due to thicker pleura and more elastic fibers in the alveolar wall [5, 16]. Consequently, the threshold for lung injury induced by ARFI in humans is anticipated to be higher than that observed in our study. Therefore, a study involving large animals is needed to assess the safety of ARFI elastography under UCA administration and investigate the risk of lung hemorrhage in humans.

## Conclusion

This study demonstrated that UCA was not a significant factor in exacerbating ARFI-induced lung hemorrhage in terms of occurrence and severity, while PRPA_0.3_ (MI_0.3_) was a significant factor regardless of UCA administration. The risks and benefits should be considered when ARFI elastography is applied to the lungs. Additionally, future studies conducted in large animals equivalent to humans under conditions similar to those in clinical settings are needed to assess the safety of combining ARFI elastography and CEUS.

## Data Availability

The data that support the findings of the study are available from the corresponding author on reasonable request.
